# An unusual case of a hypervascular solid pancreatic lesion: all that glitters is not gold

**DOI:** 10.1055/a-2885-8482

**Published:** 2026-07-07

**Authors:** Francesco Tortorici, Marco Schiavo Lena, Sara Massironi, Matteo Tacelli, Paolo G Arcidiacono

**Affiliations:** 1 PROMISE 18998 Università degli Studi di PalermoPalermoItaly; 2 Pancreatobiliary Endoscopy and Endosonography DivisionIRCCS San Raffaele Scientific InstituteMilanoItaly; 3 Pathology UnitIRCCS San Raffaele Scientific InstituteMilanoItaly; 4 Department of Medicine and SurgeryVita e Salute San Raffaele UniversityMilanoItaly; 5 Pancreas Translational & Clinical Research Center 18985 Vita-Salute San Raffaele UniversityMilanItaly

A 72-year-old woman was referred to our outpatient clinic for endoscopic ultrasound (EUS) evaluation of a small lesion in the pancreatic tail ( Video 1 ).

**Video 1** The diagnostic work-up of a hypervascular solid pancreatic lesion finally diagnosed as a metastasis of follicular thyroid carcinoma.


**Fig. 1:**
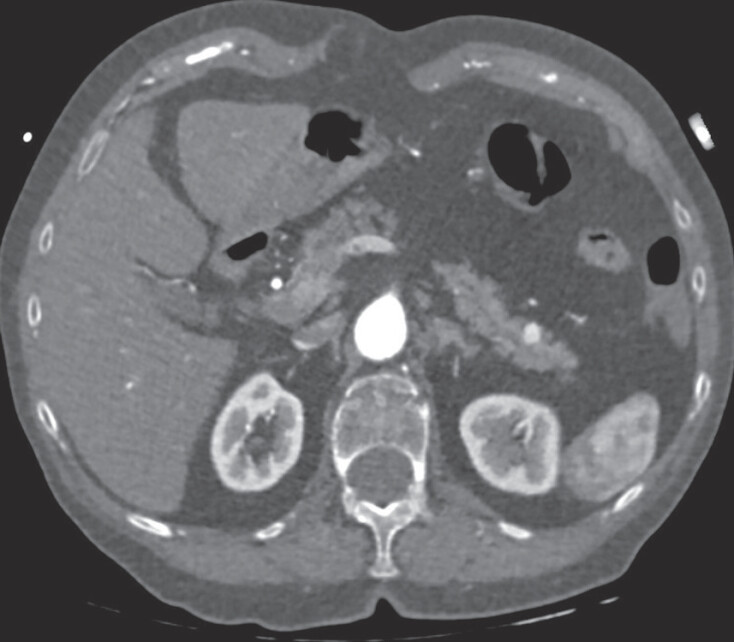
The CT scan appearance of the solid pancreatic lesion.

Previously, during cardiac surgery preoperative workup for severe mitral regurgitation requiring valve replacement, contrast-enhanced computed tomography incidentally revealed an 8 mm hypervascular round lesion ( Fig. 1 ), suspicious for a neuroendocrine tumor, in an otherwise asymptomatic patient.

**Fig. 2:**
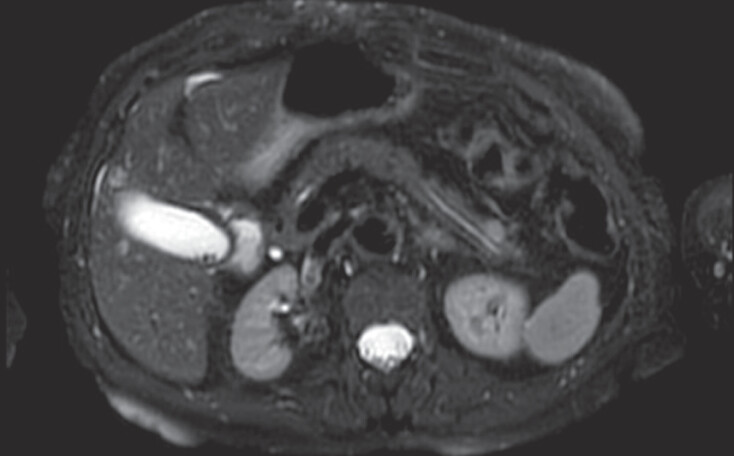
The T2-weighted MRI appearance of the solid pancreatic lesion.

Contrast-enhanced magnetic resonance cholangiopancreatography confirmed a 9-mm nodular lesion ( Fig. 2 ), mildly hyperintense on T2-weighted images, with diffusion restriction and marked postcontrast enhancement, again suggestive of a neuroendocrine tumor.

Conversely, a 68-gallium positron emission tomographic scan demonstrated only mild tracer uptake in the pancreatic tail, without significant somatostatin receptor overexpression.

**Fig 3:**
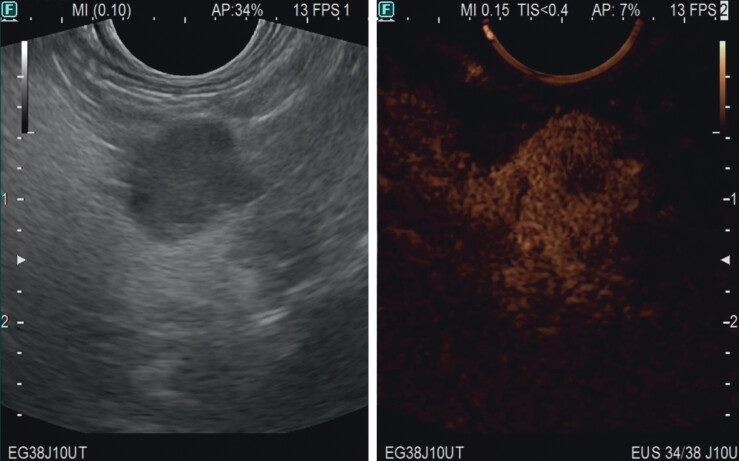
EUS hyperenhancement of the pancreatic lesion after contrast administration.

Therefore, to better clarify the nature of the lesion, we performed an EUS with needle sampling, which showed a 12×9 mm solid, hypoechoic focal lesion with mixed stiffness on elastography, hypervascularity on detective flow imaging, and avid contrast uptake after contrast administration ( Fig. 3 ).

**Fig 4:**
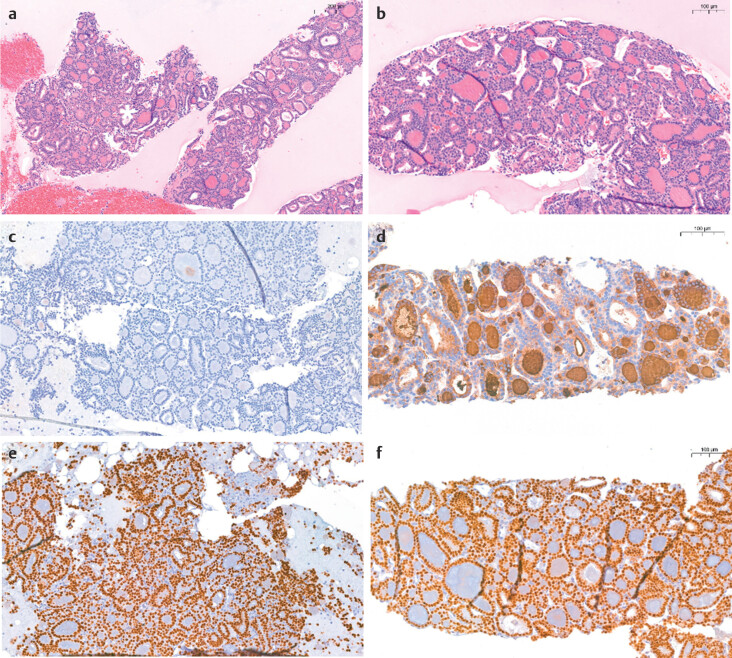
Cell block showing the presence of a well cellularized fragment, consisting of elements arranged in a follicular architecture with dense eosinophilic luminal secretion (colloid) ( **a** and **b** ). These elements resulted negative to immunohistochemical staining for chromogranin A ( **c** ), while they were positive for thyroglobulin ( **d** ), TTF-1 ( **e** ) and PAX8 monoclonal antibody ( **f** ), confirming the thyroid origin of the neoplasm.

Cytology revealed a prominent microfollicular architecture with a colloid-like material and no overt cytonuclear atypia, with strong, diffuse immunoreactivity for thyroid transcription factor-1 and paired-box gene 8 (PAX8) and negative staining for calcitonin, chromogranin A, carboxypeptidase A1, glycoprotein 2, and synaptophysin ( Fig. 4 ).

Review of the medical history disclosed thyroidectomy 15 years earlier for a solitary nodule reported as follicular adenoma in nodular hyperplasia.

These findings, together with clinical and anamnestic data, are consistent with a diagnosis of pancreatic metastasis from follicular thyroid carcinoma.

Metastases to the pancreas from follicular thyroid carcinoma are extremely rare, with only a few cases reported, and their correct characterization, as for most pancreatic lesions, requires the careful review of the patient’s clinical history, comprehensive multimodality imaging assessment, and EUS-guided cytohistological sampling for definitive histopathological diagnosis.

Endoscopy_UCTN_Code_CCL_1AF_2AZ

